# Postural Orthostatic Tachycardia Syndrome, Menopause and Hormone Replacement Therapy: Clinical Decisions in Times of Uncertainty

**DOI:** 10.3390/jcm15041477

**Published:** 2026-02-13

**Authors:** Svetlana Blitshteyn

**Affiliations:** 1 Department of Neurology, Jacobs School of Medicine and Biomedical Sciences, University of Buffalo, Buffalo, NY 14203, USA; sb25@buffalo.edu; 2 Dysautonomia Clinic, Williamsville, NY 14221, USA

**Keywords:** postural orthostatic tachycardia syndrome, hormone replacement therapy, menopause, perimenopause, hormones, estrogen, narrative review

## Abstract

Postural orthostatic tachycardia syndrome (POTS), characterized by a rise in heart rate of at least 30 beats per minute from supine to standing position without accompanying orthostatic hypotension, is one of the most common autonomic disorders with disabling cardiovascular and neurologic manifestations. Hormonal influence has been long recognized by the disorder predominantly affecting women of reproductive age, with frequent onset around menarche, exacerbation of symptoms before or during menses, and pregnancy being one of POTS triggers. Hormone replacement therapy (HRT) and menopause in women with POTS have not been studied, but issues surrounding HRT are highly relevant as women with POTS transition from reproductive age to menopause. Given a rising prevalence of POTS due to post-COVID onset and the US Food and Drug Administration recently removing the black box warning on estrogen-containing HRT formulations, informed decisions and risk assessments regarding HRT use in women with POTS are warranted. In this narrative review, existing studies on hormones and POTS and its common comorbidities are reviewed, and key points in decision-making on the use of HRT in women with POTS are discussed. In summary, for women with significant menopausal symptoms and/or exacerbation of POTS during the peri- or postmenopausal period, using some forms of HRT for treatment of menopausal symptoms may be considered, accounting for comorbidities, cardiovascular risk and other factors. Vaginal estrogen appears to be safe for most women while transdermal estrogen and micronized progesterone can be utilized for significant menopausal symptoms, although outcomes of their long-term use are unknown.

## 1. Introduction

With the United States Food and Drug Administration (FDA) removal of a black box warning from estrogen-containing products of hormone replacement therapy (HRT) in November 2025, women and their physicians have gained a powerful therapeutic arsenal in management of bothersome menopausal symptoms, such as hot flashes, mood disturbance, insomnia, fatigue and others [1]. Evidence for and against hormonal supplementation have varied greatly over the last few decades, from being considered beneficial to risky and detrimental, based on the Women’s Health Initiative (WHI) study published in 2002 [2]. Synthetic conjugated equine estrogen and medroxyprogesterone acetate used in the WHI study demonstrated increased breast cancer, stroke, thromboembolic disease and dementia risks in women taking these hormonal formulations vs. placebo, which led to a drastic fall in the use of HRT after publication of the WHI study [2]. More recently, alternative hormonal formulation consisting of low-dose oral, transdermal and vaginal estrogens and micronized progesterone became popular and widely prescribed for menopausal symptoms. While healthy women are now able to use the safer HRT formulations to improve their physical and mental health without significant concerns, peri- and postmenopausal women with medical conditions, such as POTS, chronic migraine, connective tissue disease and autoimmune disorders, and their physicians are faced with a far less clear and more complex decision-making process. In this narrative review utilizing PubMed database and studies published in English, the existing literature on hormones in POTS and its comorbidities are summarized, and considerations for clinical decision-making for the use of HRT for treatment of menopausal symptoms in women with POTS are discussed.

## 2. Perimenopause and Menopause

Perimenopause—the transitional period leading up to menopause—is characterized by fluctuating ovarian hormone production, marked variability in estrogen and progesterone levels, and an increased prevalence of vasomotor symptoms, menstrual irregularity, mood shifts, sleep disturbance, and genitourinary changes [3]. Perimenopause can be particularly challenging in women with autoimmune or connective tissue disorders, such as systemic lupus erythematosus (SLE), rheumatoid arthritis (RA), Sjögren’s disease (SjD), autoimmune thyroid disorders, systemic sclerosis, and hypermobility spectrum disorders (HSD), including hypermobile Ehlers–Danlos syndrome (h-EDS). These conditions interact in complex ways with hormonal physiology, inflammation, immune regulation, vascular integrity, and pain pathways [3].

Perimenopause encompasses a period of time during which physiologic changes lead to the final menstrual period. This phase begins with the onset of menstrual irregularities and continues until a woman reaches menopause, or one year after amenorrhea has occurred [4]. Changing hormone levels manifest in varying symptoms that can present complex clinical management situations, with up to 90% of women presenting to their physician with complaints of menopausal symptoms [5]. Traditionally, perimenopause includes an early and a late stage: the early stage is defined by occasional skipped cycles. The second stage is characterized by greater menstrual irregularity, with periods of amenorrhea lasting over 60 days and up to 12 months. Twelve or more months of amenorrhea defines the final menstrual period, after which future menses are very unlikely [4,5].

Understanding the physiology of the decline in ovarian function, which results in systemic hormonal changes and ultimately symptoms, is an important part of women’s health and patient care. It is also a critical factor in understanding brain health and the sex hormonemodulating the autonomic nervous system.

In the United States, menopause usually occurs around age 51, but anytime between age 45 to 55 is considered a normal age for menopause [6]. The diagnosis of menopause is made when a woman has not had a period for one year. Menopause occurs when the ovaries have stopped producing estrogen and the egg follicles have stopped producing other hormones as well, including progesterone, and ovulation and hormone production have ceased. Early menopause is defined as occurring before age 45, and premature menopause refers to menopause occurring before age 40 [6,7].

There is evidence demonstrating increased risk of early menopause and premature menopause in women with autoimmune disease. One study found increased risk for autoimmune diseases, especially for thyroid disorders, in women with premature ovarian insufficiency (POI) [8]. Another study found that women with POI had at least one autoimmune disorder preceding POI diagnosis 2.6 times more often compared with matched female controls, and a 2- to 3-fold risk for these diseases for several years after POI diagnosis [9]. A recent systematic review and meta-analysis found that post-menopausal women have a higher risk of developing RA compared with pre-menopausal women (odds ratio (OR) ≈ 1.35, 95% CI 1.04–1.67). The same analysis showed that early menopause (onset < 45 years) is associated with a markedly increased RA risk (OR ≈ 2.97, 95% CI 1.73–4.22) [10]. Large epidemiological data from the Nurses’ Health Study and NHS II support that menopausal status, specifically early natural menopause, is associated with increased risk of RA, especially seronegative RA, compared to pre-menopausal women [11]. In a large case–control trial of 2680 women with SjD, lower cumulative estrogen exposure in the form of earlier menopause and fewer menstrual cycles was found to be a possible risk factor for developing SjD, and higher cumulative estrogen exposure was associated with lower odds of having SjD [12]. Importantly, SjD predominantly affects middle-aged women, with majority of women having onset around perimenopause, menopause and postmenopausal age, highlighting the role of declining hormonal levels as a disease trigger [13].

## 3. Postural Orthostatic Tachycardia Syndrome (POTS)

POTS is one of the most common autonomic disorders defined by a set diagnostic criteria, which include the following: (1) an excessive increase in heart rate of more than 30 beats per minute (bpm) within 10 min of standing or during a head-up tilt table test or an increase of >40 bpm in teens up 19 years of age; (2) absence of orthostatic hypotension of at least 20 mmHg; and (3) symptoms of orthostatic intolerance that are worse with standing and typically improve upon lying down, such as palpitations, lightheadedness, and exercise intolerance, which must be present for at least 3 months [14,15]. Other conditions mimicking POTS, such as anemia, dehydration, anorexia, fever, infection, hyperthyroidism, pheochromocytoma, deconditioning, or medication side effect, need to be ruled out, although many of these conditions can be present alongside POTS [14,15].

POTS has been previously classified as neuropathic, hypovolemic and hyperadrenergic though in clinical practice these phenotypes are often overlapping rather than exclusive of each other [16]. Despite the notion that POTS has heterogeneous and diverse pathophysiologies, autoimmunity has emerged as one of its major mechanisms in the past decade. In a study of 100 patients with POTS, a higher prevalence of various non-specific autoimmune markers, including antinuclear antibodies and comorbid autoimmune disorders, was found compared to the general population [17]. Subsequently, multiple studies identified a variety of antibodies important to the autonomic nervous system and vascular control in patients with POTS, including ganglionic N-type and P/Q type acetylcholine receptor antibodies, alpha 1, beta 1 and beta 2 adrenergic antibodies, muscarinic M2 and M4 antibodies, angiotensin II type 1 receptor antibodies and opioid-like 1 receptor antibodies [18,19,20,21,22]. However, these antibodies appear to be not specific to POTS only and have also been identified in overlapping or comorbid conditions, such as myalgic encephalomyelitis/chronic fatigue syndrome (ME/CFS), small fiber neuropathy, complex regional pain syndrome and cardiovascular disorders.

POTS is a form of dysautonomia, which refers to a dysfunction or disturbance of the autonomic nervous system, and dysautonomia can be associated with a wide variety of neurologic and systemic disorders [23]. Cardiovascular manifestations of POTS suggest that while POTS is not classified as a cardiac disorder, electrophysiologic changes controlled by the autonomic nervous system and its modulation of the sino-atrial node, baroreflexes, and vascular contractility are important mechanistic factors in the pathophysiology of POTS [24]. Additionally, preload failure, hypovolemia, venous pooling and abnormal blood flow, including cerebral hypoperfusion, are major components in the complex mechanistic cascade leading to POTS, which must be considered in the context of hormonal regulation, supplementation and replacement [25].

Finally, POTS pathophysiology may involve vascular and endothelial dysfunction, hypercoagulable and pro-inflammatory state, immunologic dysregulation, mitochondrial dysfunction, blood–brain barrier disruption, mast cell hyperactivity, abnormalities in the glymphatic system, neuroinflammation, and probably other yet unidentified potential mechanisms, though some of these factors are inferred from studies on Long COVID and post-COVID POTS (Table 1) [26]. These potential mechanisms should be taken into account when considering hormonal regulation, supplementation and replacement.

## 4. POTS and Hormones

Although 80% of patients with POTS are women of reproductive age with symptom onset around menarche, pregnancy or postpartum period and with symptom exacerbation before or during menstruation and menopause, hormonal effects on POTS have not been investigated [27]. Several studies on obstetrical and gynecological issues revealed that at least half of women with POTS experience improvement during some months of pregnancy when there is substantial increase in blood volume [28]. Conversely, a majority of women with POTS recalled an exacerbation of symptoms during any point in pregnancy in an anonymous online survey [29]. During the postpartum period with hormonal drop, at least half of women experience worsening of symptoms [30]. It is unknown whether women with POTS are at a higher risk of pregnancy-induced hypertension, pre-eclampsia, eclampsia, gestational diabetes or post-partum hypertension, but clinical experience suggest that this might be the case considering that these pregnancy-related hypertensive disorders, like POTS, may be associated with sympathetic overactivity [28].

Additionally, there may be a higher prevalence of gynecologic disorders in women with POTS, such as uterine fibroids, endometriosis, PCOS and possibly a higher miscarriage rate [30,31]. This may not be surprising considering that 80% of women with POTS have at least one comorbidity and many have several, the most common being chronic migraine, HSD, and h-EDS as well as autoimmune disorders and small fiber neuropathy [27] (Table 1). These comorbidities in and of themselves can result in a number of OB/GYN manifestations and abnormalities [28].

A recent study on teens and young women with POTS with 167 participants, mean age 22 years, showed that POTS symptoms worsened prior to and during menses in the majority of women (72.4%) and that hormonal contraceptive therapy helped to control some symptoms in about half of these women [32]. The majority of those using hormonal contraceptive therapy utilized either a combined oral contraceptive or a progestin-only intrauterine device. Nearly half of patients reported dysmenorrhea as a cause for hormonal contraceptives use, with 38% using them specifically to manage POTS symptoms [32]. Ovarian cysts were reported in 20% of young women, with 10% reporting a diagnosis of polycystic ovary syndrome (PCOS) [32].

Finally, women and men with POTS have significant sexual dysfunction, including decreased libido, orgasm, sex satisfaction, and erectile dysfunction in men, implicating sex hormones as one possible factor [33]. Interestingly, significant sexual dysfunction and hormonal perturbations have been also identified in women and men with Long COVID, many of whom have dysautonomia [34]. A case series of three patients with POTS who transitioned from female to male reported improved symptoms with testosterone therapy as part of the transition [35], while male to female transitions were sometimes observed to worsen POTS symptoms in the author’s experience.

The course of POTS during the peri- and postmenopausal period has not been studied. However, three patterns are typically noted in clinical practice: 1. POTS symptoms worsen in the perimenopausal state, usually in mid-to-late 40s. 2. POTS symptoms improve in the perimenopausal and postmenopausal state, usually in mid-40s to mid-50s. 3. New-onset POTS symptoms may appear in the perimenopausal or menopausal age. Studies delineating the course of POTS in women through reproductive age, menopause and post-menopausal period are needed to understand disease course and prognostication. 

Importantly, a significant overlap between vasomotor and neurologic symptoms of menopause and symptoms of POTS results in difficulty determining the etiology and nature of these symptoms. Both menopause and POTS symptoms share a number of similar and overlapping symptoms, including flushing, increased perspiration, heat intolerance, blood pressure and heart rate dysregulation, sleep disturbance, anxiety, depression, cognitive disturbance (aka “brain fog”) and fatigue. Dysregulated blood pressure and heart rate during hot flashes of menopause may be caused by the ANS and may contribute to increased risk of cardiovascular disease in postmenopausal women [36].

## 5. POTS, Menopause and HRT

There are no data or consensus guidance on the use of HRT in patients with POTS, other autonomic disorders and comorbidities. However, given the FDA black box removal from several HRT formulations, it is expected that peri- and postmenopausal women with POTS will be asking their healthcare team to prescribe HRT [1] Additionally, many women with POTS are already taking some forms of HRT for menopausal symptoms, POTS symptoms or both. While no studies exist on the course of POTS in peri- and postmenopausal women, a significant subset of patients will experience bothersome menopausal symptoms and worsening of POTS symptoms while transitioning from monthly cycles to amenorrhea. Many menopausal symptoms overlap with POTS symptoms, such as heat intolerance, flushing, sleep disturbance, headache, joint pain and altered mood, making distinction between what is menopausal symptoms vs. POTS symptoms difficult. Since menopausal symptoms can begin up to 7–10 years before menopause, many women in their 40s or late 30s may experience worsening of pre-existing autonomic symptoms or new symptoms attributable to POTS, menopause or both. Large prospective studies are needed to determine the course of POTS, the prevalence of menopausal symptoms or, since it may be difficult to separate menopausal symptoms from POTS symptoms, worsening of POTS symptoms in the peri- and postmenopausal time frame. Furthermore, studies on the age of onset of menopause in women with POTS compared to general population in the United States are needed.

## 6. Hormones, Autonomic Nervous System and Immunology

Studies have shown that estrogen appears to modulate the ANS and that loss of estrogen, menopause, postmenopausal state and aging are associated with sympathetic overactivity [37]. In postmenopausal women, restoring estrogen via HRT has been shown to suppress muscle sympathetic nerve activity (MSNA), thereby reducing sympathetic outflow to the vascular bed [38]. A short-term trial of transdermal estradiol can reduce sympathetic nerve activity without raising blood pressure or heart rate, suggesting a potentially favorable effect on autonomic regulation [38]. More broadly, loss of estrogen in menopause is associated with worsening autonomic control, including decreased heart-rate variability (HRV), increased sympathetic dominance and less stable blood pressure, as well as baroreflex function [37]. Estrogen replacement can improve some of the negative health effects but may not be able to reverse all menopause-associated autonomic dysfunction. One study from 20 years ago using the older formulation of progestogen-containing HRT showed an increased heart rate and an attenuation of HRV in postmenopausal women [39]. Nevertheless, in otherwise healthy postmenopausal women, HRT in the form of transdermal estradiol appears to influence autonomic function toward reducing sympathetic overactivity and its favorable effects on overall health [37].

The immunomodulatory effects of estrogen are complex and dose- and tissue-dependent: estrogen may increase B-cell activity and autoantibody production but also suppress certain pro-inflammatory pathways depending on context [40]. For RA, data suggests HRT may help reduce inflammatory markers, promote bone remodeling toward bone preservation via IGF-1 and protect bone density [41]. Nevertheless, a large 2025 population-based study found that HRT use preceding the diagnosis was associated with higher odds of developing new-onset lupus or systemic sclerosis [42]. This raises the possibility that exogenous hormones could, potentially, contribute to disease onset or at least coincide with early disease manifestations, though causality is not established, and confounding factors are possible.

With respect to connective tissue, estrogen may help preserve collagen and tissue integrity in skin, bone and discs, which could be beneficial for women with connective tissue disorders, such as HSD and h-EDS, but the associated decrease in tendon and ligament stiffness could also worsen joint laxity and instability [43,44]. These estrogen effects are important to consider in disorders characterized by hypermobility or abnormal connective tissues, such as HSD and h-EDS.

Finally, estrogen can directly activate mast cells via non-genomic estrogen receptor-α and calcium influx. In one study, physiological concentrations of 17-β-estradiol caused partial degranulation with release of β-hexosaminidase from cultured mast cells, including human-derived cells, even without allergen and IgE stimulation. The mechanism involved a non-genomic estrogen-receptor–α on the mast cell membrane, producing a calcium influx that triggered mediator release [45]. The study showed that estrogen also potentiated IgE-dependent degranulation and increased leukotriene C4 production [45]. More broadly, review of mast cell biology demonstrates that mast cells express receptors for a variety of triggers, including hormones [46]. In contrast, progesterone may have inhibitory or stabilizing effects on mast cells [46]. Taken together, there is strong biological plausibility that exogenous or endogenous sex hormones, especially estrogen, can modulate mast cell activity, potentially triggering degranulation or lowering the threshold for activation.

## 7. HRT and Cardiovascular Disease

A number of studies have shown that oral estrogen–progestin therapy may be associated with an increased risk of heart disease and venous thromboembolism [47]. However, this effect may be reduced by transdermal estrogen, which is considered to have a better safety profile than oral estrogen because it does not pass through the liver and does not induce pro-coagulation pathways. Observational studies suggest that transdermal estrogen does not increase venous thromboembolism above baseline, and that low-dose transdermal estrogen has the most favorable side effect profile. One study did find a potential cardiovascular benefit when HRT was started close to the menopausal onset, but these results have not been replicated in larger studies [48]. Therefore, the current consensus is that HRT use is not recommended for primary or secondary prevention of cardiovascular disease, which is the consensus across several major medical societies [49]. However, HRT may be initiated in healthy women with normal body mass index, glucose and lipid level who are less than 60 years old or less than 10 years postmenopause to treat menopausal symptoms [49,50]. Women with high cardiovascular risk or a personal history of heart disease, thromboembolism, clotting disorders, diabetes, vascular aneurysms, chronic kidney or liver disease or estrogen-sensitive cancers should consider avoiding systemic HRT due to its potential risks [49].

## 8. Decision-Making on HRT in POTS

Since there are no studies on use of HRT in women with POTS and there are no longitudinal studies on long-term use of transdermal estrogen and micronized progesterone in healthy women or women with pre-existing medical conditions, the clinical decision process is difficult, imprecise, nuanced, and fraught with assumptions and hypothetical benefit vs. risk estimations. In the absence of randomized controlled trials and until robust data become available, clinical decision-making needs to be inferred or extrapolated from the limited data that exists on hormonal supplementation in women with health conditions, such as cardiovascular disease, autoimmune disorders and neurologic conditions. In addition, individualized approach and patient-centered decision-making need to be emphasized. Shared decision-making with the patient should utilize the patient’s symptoms of POTS and menopause, comorbidities, diagnostic markers of autoimmunity, metabolic and hypercoagulable state, and a family history of cardiovascular and thromboembolic disorders, stroke and hormone-sensitive cancers.

Importantly, it is unknown whether women with POTS are at risk for postmenopausal cardiovascular disease, hypertension, stroke and metabolic syndrome compared to the age- and sex-matched general population. It is possible that there may be a higher risk of these health conditions than in the general population given the sympathetic overactivity with its resultant pro-inflammatory state. Second, the presence of comorbidities such as h-EDS/HSD, chronic migraine, autoimmune disorders and small fiber neuropathy may add further concerns with respect to cumulative risk for cardiovascular, thromboembolic and cerebrovascular disease over time, especially when this risk increases for healthy postmenopausal women. Longitudinal studies on postmenopausal women with POTS are lacking but are urgently needed to assess their cardiovascular and stroke risk compared to postmenopausal women in the general population and to develop data-driven counseling and heart and brain disease prevention strategies in this patient population.

As is the case with many aspects of POTS care with a lack of data, the decision whether to start HRT or not for menopausal symptoms should be individualized and patient-centered. For example, if there are significant personal cardiovascular risks, oral estrogen and progesterone may be avoided, but vaginal estrogen is likely safe to use, and transdermal estrogen may be considered, though even long-term transdermal estrogen use in women with high cardiovascular risk is currently not recommended [49]. Women with chronic migraine, particularly with migrainous aura, in whom the risk of stroke is elevated above the general population, might also aim for vaginal estrogen and possibly short-term transdermal estrogen use if menopausal symptoms are severe and if the patient prefers to use HRT. Women with POTS and autoimmune disease, especially APS and lupus, may be at a higher risk for cardiovascular and thromboembolic disease and therefore would need to minimize systemic estrogen exposure. To this end, vaginal estrogen might be acceptable for genitourinary symptoms of menopause, especially if accompanied by overactive bladder and frequent urinary tract infections. All autoimmune disorders carry a higher risk of thromboembolic disease [51], which may necessitate reduced systemic exposure to estrogen and synthetic progesterone though the effects of long-term micronized progesterone use are unknown. Vaginal estrogen, short-term transdermal estrogen and micronized progesterone might be used instead of oral HRT to minimize the risk of thrombosis. Review of HRT in women with various autoimmune disorders is beyond the scope of this review, and long-term studies are needed to determine the risks and benefits of various HRT types in each autoimmune disorder.

Additionally, observational data suggest that HRT use is associated with a higher prevalence of migraine headaches compared with nonusers (OR 1.42, 95% CI 1.24–1.62) [52]. The risk is especially prominent with oral estrogen: oral estrogen increases activation of hepatic coagulation factors, which raises the risk of venous thromboembolism and potentially stroke [53]. For women with migraine with aura, the concern is dual: first, migraine with aura is itself an independent risk factor for ischemic stroke, and second, exogenous estrogen, especially in oral form, may further increase that risk [54]. However, transdermal estrogen appears to have a more favorable vascular safety profile, and observational data suggest a lower risk of ischemic stroke with transdermal estradiol than oral estrogen in postmenopausal hormone therapy [53,54]. Given the risks, dose, route and timing since menopause, individual risk factors must be carefully evaluated when considering HRT in women with POTS and comorbid migraine [52,53,54,55].

Ultimately, a personalized approach and a discussion between the patient and their physician are needed to determine the possible risks vs. benefits prior to initiating HRT. This decision can utilize the patient’s menopausal symptoms, their effects on POTS, comorbidities, autoimmune markers, cardiovascular risks and personal and family history of stroke, heart disease, thromboembolic disease, dementia, breast cancer and osteoporosis. Additionally, hydration status and mobility issues may be considered for patients with severe POTS, especially with comorbid ME/CFS. When HRT cannot be used because it is contraindicated, the risks are too high, or because the patient prefers not to use it, non-HRT therapies for menopausal symptoms should be tried (Table 2) [56].

Combining the limited data on HRT use in peri- and postmenopausal women with medical conditions, the following key points emerge as reasonable considerations in women with POTS despite the uncertainty:

- Vaginal estrogen (0.01% estradiol cream) 1 gm weekly can be used in most women with POTS due to its negligible to minimal systemic absorption for genitourinary symptoms and prevention of urinary track infections.
- Transdermal estrogen patch can probably be used in many women with POTS for vasomotor symptoms, but its long-term use adverse effects in postmenopausal healthy women and women with autonomic dysregulation are unknown.
- Micronized progesterone can probably be tried in most women with POTS for menopause-related insomnia and anxiety, but its long-term use effects in postmenopausal healthy women and women with autonomic dysregulation are unknown.
- Low-dose oral estrogen can be considered in some women with POTS in the peri- or postmenopausal stage who experience severe menopausal symptoms and have low risks for cardiovascular and thromboembolic disease, stroke and breast cancer.
- Low-dose testosterone cream can be considered on a case-by-case basis in peri- and postmenopausal women with POTS and hypoactive sexual desire disorder, but its use has not been studied in women.
- Non-hormonal management of menopausal and autonomic symptoms is encouraged, including adjusting current pharmacologic and nonpharmacologic therapies for POTS and implementing new non-hormonal treatment options for symptoms, such as hot flashes, blood pressure dysregulation, insomnia, pain and mood disturbance (Table 2). For example, beta blockers can help with flushing— associated tachycardia and hypertension. Clonidine patches or tablets can reduce sympathetic overactivity, blood pressure spikes, hyperhidrosis and insomnia. Fezolinetant and elinzanetant are non-hormonal oral medications that work on neurokinin pathways in the brain that control temperature, which have been FDA-approved for menopausal hot flashes.
- Close monitoring of heart rate and blood pressure, as well as menopausal and POTS symptoms, is encouraged before and after initiation of HRT formulations beyond vaginal estrogen, which likely does not require any monitoring, given its negligible to minimal systemic estrogen absorption.

## 9. Future Direction

Multicenter randomized controlled trials of vaginal estrogen, transdermal estrogen and micronized progesterone—currently widely used hormonal formulations—are needed to determine long-term outcomes, benefits and risks of HRT, not only in healthy peri- and postmenopausal women but also in women with POTS, other forms of dysautonomia, autoimmune disease, chronic migraine and other neurologic disorders. Menopausal symptoms can be severe in some women and greatly impair physical and mental health as well as quality of life and functional status, so recognition and management of these symptoms is critical to women’s health. Disorders, such as POTS and other chronic medical conditions, present a special challenge and should necessitate additional considerations and a personalized approach when discussing and prescribing HRT. The benefits of replacing hormones on overall cardiovascular health and brain health as part of preventative medicine also need to be explored [57].

Future studies should investigate the following important questions:

1. Is menopausal and perimenopausal age similar in women with POTS to women in the general US population?
2. Is the course of POTS improved, worsened or unchanged by menopause?
3. Do women with POTS and dysautonomia have a higher incidence rate of hypertension, cardiovascular disease, stroke and metabolic syndrome than women in the general population given decades of sympathetic overactivity, cerebral hypoperfusion, hypovolemia and possible pro-inflammatory state?
4. Is HRT in the form of transdermal estrogen and micronized progesterone safe for the majority of peri- and postmenopausal women with POTS?
5. What factors specifically related to POTS should be considered in the risk assessment in clinical decisions for or against HRT use?
6. Are the risks and benefits of HRT comparable in peri- and postmenopausal women with POTS vs. women without POTS?
7. Can extremely low-dose testosterone supplementation be safely used to treat not only hypoactive sexual desire disorder but also symptoms of POTS in pre-, peri- and postmenopausal women?

## 10. Conclusions

There are no data to recommend for or against HRT use in peri- or postmenopausal women with POTS, which creates uncertainty for both physicians and patients. Multiple factors, including sympathetic overactivity, cerebral hypoperfusion and possible pro-inflammatory state and association of POTS with migraine, connective tissue abnormalities, mast cell hyperactivity and autoimmune disorders, suggest that concerns over the use of HRT are valid, requiring careful consideration and personalized risks vs. benefits assessment. Limited data on women with other health conditions, along with clinical experience, suggest that women with significant menopausal symptoms and/or exacerbation of POTS during the peri- or postmenopausal period may utilize some forms of HRT for symptomatic treatment, accounting for comorbidities, cardiovascular risk, autoimmune markers and personal and family history of stroke, heart disease, thromboembolic disease and hormone-sensitive cancers. In general, vaginal estrogen appears to be safe for use in most women while transdermal estrogen and micronized progesterone may be utilized for significant menopausal symptoms, although at this time, outcomes of their long-term use, both in healthy women and women in POTS, are unknown.

## Figures and Tables

**Table 1:** Common comorbidities and pathophysiologic factors of POTS.

**Comorbidities**	Migraine with and without aura Hypermobility spectrum disorders and hypermobile Ehlers–Danlos syndrome Mast cell activation syndrome Autoimmune disorders • Hashimoto’s thyroiditis • Sjogren’s disease • Anti-phospholipid syndrome • Celiac disease • Rheumatoid arthritis • Lupus • Undifferentiated connective tissue disease Small fiber neuropathy Sleep disturbance
**Pathophysiologic Factors** **(Established and Proposed)**	Sympathetic overactivity Parasympathetic underactivity Hypovolemia Altered vascular resistance Venous disease with vascular compression syndromes Preload failure Beta adrenergic sensitivity Autoimmunity Immune dysregulation Endothelial dysfunction Connective tissue abnormalities Mitochondrial dysfunction Hypercoagulable state Blood–brain barrier disruption Glymphatic system alterations Mast cell hyperactivity Cerebral hypoperfusion Neuroinflammation

**Table 2:** Hormonal and non-hormonal therapies for menopausal symptoms in women with POTS.

**Hormonal**	**Transdermal estrogen patch**	short-term for symptomatic relief
	**Vaginal estrogen**	may be long-term for genitourinary symptoms and/or frequent UTI
	**Micronized progesterone**	short-term for new-onset insomnia or anxiety related to perimenopause or menopause or long-term, but long-term effects are unknown
**Non-hormonal**	**Beta blockers**	atenolol, metoprolol, bisoprolol, propranolol
	**Sympatholytics**	clonidine patch or tablets, guanfacine, methyldopa
	**Neuropathic**	gabapentin, low-dose naltrexone, SSRIs
	**Antihistamines**	loratadine, cetirizine, fexofenadine, famotidine, hydroxyzine, ketotifen, diphenhydramine
	**Neurokinin receptors antagonists**	fezolinetant, elinzanetant
	**Sedatives/hypnotics**	**Supplements:** melatonin, magnesium, Valerian root, vitamin D3, CBD **Medications:** doxepin, trazadone, mirtazapine, zolpidem, ramelteon, clonazepam
	**Non-pharmacologic**	fluids 3 L/day, sodium chloride, gluten-free, low-carb, low-histamine diet, reduced caffeine intake, no alcohol, breath work, meditation, cognitive behavioral therapy, supine and sitting exercise, compression garments, cooling vest and blanket, non-invasive vagus nerve stimulation, sleep hygiene

## Data Availability

No new data were created or analyzed in this study. Data sharing is not applicable.
